# What is the nature of cache memory in Parids? A comment on Chettih et al. 2024

**DOI:** 10.1007/s10071-025-01932-7

**Published:** 2025-02-12

**Authors:** Tom V. Smulders, Sen Cheng

**Affiliations:** 1 School of Psychology, Newcastle University, Newcastle upon Tyne, UK; 2 Institute for Neural Computation, Ruhr University Bochum, Bochum, Germany

**Keywords:** Food-hoarding birds, Chickadee, Paridae, Recollection, Familiarity, Cued recall, Free recall

## Abstract

Recent findings by Chettih et al. (Cell 187: 1922–1935, 2024) from electrophysiological recordings in the hippocampus of black-capped chickadees shed light on the debate about how food-hoarding Parids may remember their cache sites. When birds retrieve caches, a “bar code” is reactivated, which is very similar to the code generated when the same cache was made. The current evidence suggests that this bar code is only triggered after the bird starts to retrieve the cache, and not in anticipation. This finding is more consistent with cued recall than with free recall of cache locations.

It has been established for over 40 years now that food-hoarding titmice and chickadees (Paridae) can use memory to retrieve their caches (Sherry et al. 1981; Shettleworth and Krebs 1982), and just over 35 years that the hippocampus is required for this retrieval (Sherry and Vaccarino 1989). However, there is a debate in the literature whether this memory is retrieved by free recall of the caching event (Pravosudov 2023), or by a feeling of familiarity upon viewing the cache site (Smulders et al. 2023; Smulders and Read 2024). A recent study recording electrophysiological activity from populations of single neurons in the black-capped chickadee (*Poecile atricapillus*) hippocampus may shed some light on this question (Chettih et al. 2024).

The main finding of Chettih et al.’s (2024) study is that populations of hippocampal neurons contain, in addition to spatial representations of where the bird is in space, a representation (a combination of firing patterns of many neurons they term a “bar code”) activated when caches are made (Chettih et al. 2024). These bar codes are unique to each caching event, but they are very similar between two sequential caching events at the same site (after about 10 min, this similarity is independent of the time elapsed between the two events). This similarity with the bar code of the first caching event wanes with each next cache made at the same site. Critically, the bar code is reactivated at the time the cache is retrieved. The similarity in bar codes between a caching event and its associated retrieval event is roughly equal to the similarity between one caching event and the next caching event in the same site. However, the bar code during the retrieval event is much more similar to the immediately preceding caching event, than to the other caching events at the same site. The bar code is also reactivated during checks (events during which the cache site is briefly opened, but nothing is added or taken out), but the similarity to the caching bar code is much lower than that between consecutive caching event bar codes or between the bar codes of a caching event and its associated retrieval event. Interestingly, the evidence presented in the paper only shows the bar code to be activated after the bird arrives at the cache site, not just during the caching episode, when presumably the memory is being encoded, but also during checks and retrievals, when you might expect memories to be retrieved. How do these findings shed light on the nature of the cache memory?

Firstly, the decay in the similarity of the bar code during checks before the cache is retrieved suggests that checks are not refreshers of memory (Roth et al. 2012), akin to “re-consolidation” of memories (Abel and Lattal 2001) to improve memory retention. If that was the case, we would expect the bar code similarity to the caching event to become more consistent with repeated checks. The bar code similarity during subsequent checks of a full cache site does remain above “place cell” baseline, however, so some extra information is retained over and above just spatial information. However, when the bird eventually retrieves the cache, the retrieval bar code is much more similar to the caching bar code than to neural activity during the checks. This suggests that there is information being activated during retrieval that is not activated during checks, possibly related to the exact details of the caching event, such as the content of the cache (Sherry 1984), or how recently the cache was made (Feeney et al. 2009).

Secondly, the timing of when the bar code is re-activated suggests that this code is not used to navigate towards a remembered cache site (Chettih et al. 2024). If the bar code represents a memory for the caching event, this pattern suggests that the bird may not remember that it has cached something in that site until after it lands on the site. However, this interpretation may be an artifact of the way the data were collected and analysed. The birds cached and retrieved in a 76 × 76 cm arena, with 128 cache sites spaced by a minimum of 5.3 cm. This is very close together compared to the field, where, when marsh tits (*Poecile palustris*) cache from a single food source, nearest neighbour distances tend to be a minimum of 2 m, and probably even further for naturally hoarded food (Brodin 1992; Cowie et al. 1981). The small dimensions of the caching environment may influence when memories are reactivated. Also, the authors analyse the similarities between the bar codes by aligning the neural activity to the arrival at the cache site and averaging across pairs of events. It is, however, possible that the bar code is activated before the bird arrives at the cache site, but that the timing of this activation varies from event to event. In that case, averaging across event pairs would not detect such a reactivation. In order to detect it, the authors would need to conduct a search for a peak in similarity to the bar code for each individual retrieval event, covering a fairly long time interval leading up to the arrival (e.g. 20 seconds back), akin to the analysis used to detect neural replay during sleep in the rat hippocampus (Skaggs and Mcnaughton 1996) or the song system (Dave and Margoliash 2000). As Chettih et al. (2024) point out themselves, only if there is evidence for bar codes being activated before the birds arrive at the site could they potentially contribute to free recall of caching events. This would especially be the case if the code was reactivated at a time when the bird was not looking at the cache site, as in the small environment in which the study was run, all sites are in principle visible from everywhere in the environment. Such an analysis remains to be carried out.

If such an analysis were to show that there is no pre-activation of the bar codes, or that bar codes are only activated when the bird is directly looking at the cache site, then that would argue against a free recall mechanism of memory retrieval. Which memory mechanism would be involved then? Smulders et al. (2023) analysed cache retrieval by coal tits (*Periparus ater*) by applying the dual-process model of recognition memory to Receiver Operating Characteristics (ROC; Yonelinas 1994) curves derived from the cache retrieval performance. In humans, this analysis allows for an estimation of the relative contributions of familiarity and recollection to recognition memory. Familiarity is the feeling of having encountered a stimulus before, but without any context of where or when this happened, while recollection includes recall of contextual information about the event at which the stimulus has been previously encountered (Yonelinas 1994). In the ROC analysis, the Y-intercept of the ROC curve represents the process of recollection (free recall) and the curvilinearity of the curve indicates familiarity. However, the coal tit analysis did not show any significant Y-intercept, leading to the conclusion that hoarding birds mainly use familiarity, rather than recollection in recognizing cache sites for retrieval. Familiarity requires the birds to see the cache site (possibly from a distance) before the recognition of the site can be triggered. Smulders et al. (2023) argue that this fits the ecology of these birds, who hoard in the same types of places where they regularly forage. Rather than targeting a particular cache site for retrieval, it would then be possible that they only remember a cache site when they come near (i.e. in view of) one while foraging. Such a mechanism is more efficient than navigating to a cache site from far away.

However, Chettih et al. (2024)’s results are only partially consistent with a familiarity mechanism. The bar codes associated with one particular cache site are much more similar to each other than to those associated with other cache sites. This kind of similarity might be expected from a familiarity-based memory process. However, on top of the similarity between caching events at the same site, each pair of caching and retrieval events are still more similar to each other than to other pairs of events at the same site. This suggests that, possibly in addition to the familiarity-based mechanism, a unique event recollection mechanism may also be at work. So why did Smulders et al. (2023) not find evidence of a recollection process? Firstly, it should be emphasized that some coal tits did show a high Y intercept (i.e. potential sign of recollection) on their individual ROC curves for some trials, but these were averaged out across birds. Smulders et al. (2023) therefore did not completely rule out the contribution of recollection but emphasized the importance of familiarity. Secondly, there are alternative interpretations of the lack of Y-intercept in ROC curves than a memory mechanism that is based purely on familiarity and not on recollection, as suggested by the dual-process model (Yonelinas 1994). Hakobyan and Cheng (2021) built a computational model that only contains a single underlying memory process: cued recall (i.e. neither free recall nor familiarity). Using recognition ROC curves to plot the performance of the model, they were able to show that if the to-be-remembered information was very similar to each other, resulting ROC curves would have a Y-intercept of zero. Since cache sites in typical cache retrieval studies are usually close in space and visually very similar to each other (Chettih et al. 2024; Sherry 1984; Sherry et al. 1981; Shettleworth and Krebs 1982; Smulders et al. 2023), cued recall could account for zero Y-intercept of the recognition ROC curve in caching. Taken together, Smulders et al. (2023) and Chettih et al. (2024)’s findings therefore point to the use of cued recall, in which the birds need to be exposed to the cache site in order to trigger the memory that something is hidden there. Cued recall is known to be dependent on the hippocampus in rats (Langston and Wood 2008) and would allow for recall of the unique caching event, including the wider context in which it occurred, as would be needed for retrieval of what-where-when memory of caches (Feeney et al. 2009).

In conclusion, the recent results by Chettih et al. (2024) and Smulders et al. (2023) suggest that food-hoarding birds may well recollect specific hoarding events, but that those memories might need to be triggered by the perception of the cache site, through a process of cued recall. Such a cued recall mechanism would be a very efficient memory retrieval strategy in the context of Parid caching ecology. The results cannot rule out free recall mechanisms, however. Future studies should be designed to explicitly test for the existence of free recall memory mechanisms, possibly by searching for earlier reactivations of the bar codes, ideally when the bird is not looking at the cache site.

## Data Availability

No datasets were generated or analysed during the current study.
