# Correction: What is the nature of cache memory in Parids? A comment on Chettih et al. 2024

**DOI:** 10.1007/s10071-025-01988-5

**Published:** 2025-07-25

**Authors:** Tom V. Smulders, Sen Cheng

**Affiliations:** 1 School of Psychology, Newcastle University, Newcastle upon Tyne, UK; 2 Institute for Neural Computation, Ruhr University Bochum, Bochum, Germany

**Correction: Animal Cognition (2025) 28:13**

10.1007/s10071-025-01932-7

In this article the ‘Conflict of Interest’ statement was missing and should have been “Editor T. V. Smulders is a guest editor for the journal and has not taken part in the manuscript’s peer review process”. The original article has been corrected.

